# Image Quality and Patient-Specific Organ Doses in Stone Protocol CT: A Comparison of Traditional CT to Low Dose CT with Iterative Reconstruction

**DOI:** 10.1155/2018/5120974

**Published:** 2018-09-27

**Authors:** Raghav Pai, Rishi Modh, Rebecca H. Lamoureux, Lori Deitte, David C. Wymer, Anna Mench, Izabella Lipnharski, Carl Henriksen, Manuel Arreola, Benjamin K. Canales

**Affiliations:** ^1^Department of Urology, University of Florida, Gainesville, FL, USA; ^2^Department of Radiology, University of New Mexico, Albuquerque, NM, USA; ^3^Department of Radiology, University of Florida, Gainesville, FL, USA; ^4^Division of Clinical Radiological Physics, University of Florida, Gainesville, FL, USA

## Abstract

**Objective:**

To compare organ specific radiation dose and image quality in kidney stone patients scanned with standard CT reconstructed with filtered back projection (FBP-CT) to those scanned with low dose CT reconstructed with iterative techniques (IR-CT).

**Materials and Methods:**

Over a one-year study period, adult kidney stone patients were retrospectively netted to capture the use of noncontrasted, stone protocol CT in one of six institutional scanners (four FBP and two IR). To limit potential CT-unit use bias, scans were included only from days when all six scanners were functioning. Organ dose was calculated using volumetric CT dose index and patient effective body diameter through validated conversion equations derived from previous cadaveric, dosimetry studies. Board-certified radiologists, blinded to CT algorithm type, assessed stone characteristics, study noise, and image quality of both techniques.

**Results:**

FBP-CT (n=250) and IR-CT (n=90) groups were similar in regard to gender, race, body mass index (mean BMI = 30.3), and stone burden detected (mean size 5.4 ± 1.2 mm). Mean organ-specific dose (OSD) was 54-62% lower across all organs for IR-CT compared to FBP-CT with particularly reduced doses (up to 4.6-fold) noted in patients with normal BMI range. No differences were noted in radiological assessment of image quality or noise between the cohorts, and intrarater agreement was highly correlated for noise (AC2=0.873) and quality (AC2=0.874) between blinded radiologists.

**Conclusions:**

Image quality and stone burden assessment were maintained between standard FBP and low dose IR groups, but IR-CT decreased mean OSD by 50%. Both urologists and radiologists should advocate for low dose CT, utilizing reconstructive protocols like IR, to reduce radiation exposure in their stone formers who undergo multiple CTs.

## 1. Introduction

Kidney stones affect 1 in 11 people in the US and account for 1.5 million emergency department visits annually [1, 2]. CT is the gold standard imaging modality for stone diagnosis during renal colic episodes due to its high sensitivity and specificity. Recurrent stone formers also undergo surveillance CT scans to monitor for stone growth and drug therapy effects [3]. Not surprisingly, the rising number of CT studies has increased the concern of long term radiation dose effects in this population, as the widely accepted linear-no-threshold model predicts a steady increase in the probability of developing cancer with even the smallest amount of radiation dose [4].

For decades, the conventional filtered back projection (FBP) reconstruction algorithm has been used due to the quick nature of reconstructing cross-sectional images from tomographic projections. FBP is not model-based, meaning that no data processing software is used to account for reconstruction modeling characteristics and therefore requires a higher number of photons (higher dose) to maintain an appropriate level of noise. In 2011, iterative reconstruction (IR) was made available in modern CT scanners. Compared to FBP, the model-based nature of this reconstruction algorithm inherently decreases noise, allowing for lower counting statistics and radiation dose to achieve similar or even reduced noise in the final image.

To date, three groups have evaluated the use of IR in CT specifically for the diagnosis of stone disease [5–8]. Although informative, these studies did not have appropriate image quality assessors, did not enroll sufficient patient number, and did not attempt a quantitative assessment of dose savings by patient size or organ specific doses [5–8]. The purpose of this study is to describe and quantify radiation dose-reduction according to patient body size while comparing subjective image quality using validated formulas between FBP-CT and IR-CT acquisition techniques in kidney stone formers.

## 2. Materials and Methods

With IRB approval, noncontrasted CT exams of the abdomen and pelvis over the course of one calendar year from our institution were analyzed for the presence of stones. The exams from two iterative reconstruction capable CT units (Aquilion ONE and AquilionVision, Toshiba America Medical Systems, Tustin, CA) and four FBP-only capable CT units (Aquilion 16 and Aquilion 64, Toshiba America Medical Systems, Tustin, CA) were included in the study. Patients were randomly assigned to the scanners based on CT unit availability during their clinical course (in-patient, ED, etc.). To limit bias in CT unit use, only scans from days when all six scanners were functioning were included in the study. All imaging was performed at 120 kVp with 5 mm slice thickness and tube-current modulation (SURExposure, Toshiba America Medical Systems, Tustin, CA). IR-CT studies used Adaptive Iterative Dose Reduction 3D (AIDR 3D, Toshiba America Medical Systems, Tustin, CA) with dose index settings at manufacturer recommendations. Technical parameters such as axial coverage, collimation, and X-ray detector performance were previously set by radiologists and physicists at our institution to optimize the reconstruction algorithm used.

### 2.1. Clinical and Radiological Data

Using the speech recognition platform Powerscribe 360® (Nuance Industries, Burlington, MA), CT dictations were searched to find the word “stone” and included only exams identified by radiology to have a stone within the urinary tract. Clinical data was then captured, including body mass index (BMI), age, race, gender, reason for scan, patient hospital status (inpatient, outpatient, and ED), and the previous number of abdominal CT scans obtained at our institution over the past five years. Patients were excluded if BMI (height or weight) was missing from the chart.

Two board-certified radiologists (EV = 15 years' experience; DCW = 25 years' experience), blinded to CT algorithm type and previous dictations, reviewed each patient's image series for stone characteristics, including total stone burden (mm^2^), size of largest stone, degree of hydronephrosis, total stone number, and stone location. Reviewers were asked to assess study noise using a 3-point scale (1 = minimal; 2 = acceptable; 3 = excessive) and diagnostic study quality/confidence for making the diagnosis using a 5-point Likert scale (1 = poor/diagnostically unacceptable; 2 = suboptimal; 3 = acceptable; 4 = good; 5 = excellent). When measurements differed, a third board-certified radiologist served as an arbitrator.

For the radiation dose calculations (described further in the following section) the patient's anterior-posterior diameter and lateral diameter were obtained by a radiologist at the midsection of the scan range. The machine produced Volumetric CT Dose Index (CTDI_VOL_) correlating to each exam was also recorded for use in the organ dose calculations. Clinical data collectors, radiologists, and medical physicists were blinded to each other's results. All data were stored on a secure server (REDCap Software™, Version 6.5.3).

### 2.2. Radiation Dose Calculations

Medical physicists calculated absorbed radiation doses for the organs contained entirely within the scan range. This included skin, liver, stomach, small intestine, and colon for both males and females and ovary/uterus for females only. Organ doses were performed utilizing locally validated, linear formulas previously derived from empirically measured (dosimeter studies), absorbed radiation doses completed in postmortem subjects within the same scanners [9, 10]. In addition to requiring scan CTDI_VOL_, these formulas utilize anterioposterior and lateral diameter of each patient to determine the effective body diameter (EBD). EBD is an established patient-size metric that translates the elliptical area of the patient's cross section into a circle of equal area to be given a singular diameter (see Supplemental Figure 1). Once EBD was calculated, organ doses were calculated for each individual patient, assuming that each organ was completely within the X-ray beam on the clinically utilized scan range for the stone protocol CT (kidney/ureter/bladder).

### 2.3. Statistical Analysis

To detect an effect size of ≥ 0.2 at *α* = .05 and power of 85% between the two groups, a total sample size of 300 was estimated to be required. Descriptive data between groups were calculated as means with standard deviation (STD) and compared using paired Student's* t*-test. Two-tailed Fisher's exact test and ANOVA were used for categorical variables, and statistical significance was defined as p <0.05. Agreement among image quality and disease assessment evaluators was calculated using a second-order agreement coefficient (Gwet's AC2 statistic). All statistical analyses were performed using Statistical Analysis Software (SAS) 9.4 (SAS Institute Inc., Cary, NC).

## 3. Results

Over a one-year period, a total of 4,735 patients had noncontrasted abdominal CTs at our main institution campus. Of these, 2,124 had the word “stone” in their CT dictation including 340 patients who met inclusion criteria and had urinary tract stones (250 FBP-CT and 90 IR-CT). Patient cohorts were similar in demographics and imaging history (Table 1) with no identifiable differences in gender, age, BMI, or race. Both cohorts were obese (mean BMI = 30.3) and had undergone an average of two prior abdominal and pelvic CT exams at our institution over the previous five years (Table 1).

The majority of identified stones were small (Table 2, mean 5.4 ± 1.2 mm), located within the kidney (75%), and single in morphology (88%) with no statistically significant differences noted between groups. Ureteral stones (mean 4.6 ± 0.9 mm) were smaller than renal stones (mean 5.7 ± 1.2 mm), and a larger proportion of patients with ureteral stones underwent IR-CT compared to FBP-CT (31% vs. 22%, p=0.07). Distribution of hydronephrosis and bladder stone location was also found to be similar between groups. Using 3- and 5-point scales, no difference was seen in CT noise or quality (Table 2) between groups, and only 3 (0.9%) image series were found to be less than acceptable (one IR-CT and two FBP-CT). Intrarater agreement was highly correlated for noise (AC2=0.873) and quality (AC2=0.874) between blinded radiologists.

**Figure 1:**
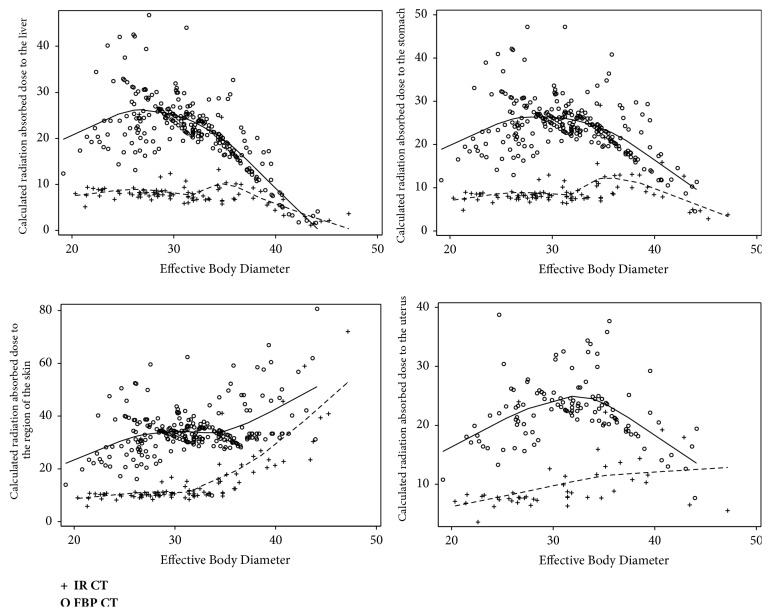
Organ-specific radiation doses to liver, stomach, skin, and uterus (female patients only) calculated for two different patient cohorts utilizing FBP-CT or IR-CT and based on effective body diameter measurements. Mean FBP-CT dose was 2-3-fold higher than IR-CT dose with highest dose savings for patients size between 25 cm and 32 cm EBD.

Mean organ-specific dose (OSD) was 54-62% lower across all organs for IR-CT compared to FBP-CT.  Table 3 shows the mean ± standard deviation. Notably, smaller and mid-sized EBD patients (20 cm to 32 cm) on average saw larger dose savings than large EBD patients. The average IR-CT delivered 36% of the dose of a FBP-CT to small and mid-sized patients; large patients scanned with IR-CT on average received 55% of the dose FBP-CT produced.  Figure 1 demonstrates the variation in dose savings for varying EBD as well.

## 4. Discussion

Although there is increasing literature to promote use of ultrasound, CT continues to be the gold standard for the diagnosis of urinary stones [11, 12]. The use of abdominal CT for kidney stone diagnosis has raised concerns over patient radiation exposure [13], and cumulative radiation doses associated with repeated kidney stone CT examinations have been reported to range from 8.5 mSv to as high as 154 mSv in this population [14]. Our cohort of urinary stone formers had an average of 2-3 CTs over the five-year study review period at our institution alone, not including plain films, fluoroscopy, outside studies, or the current CT evaluated by our group. Reducing radiation CT exposure in this population is crucial to reduce lifelong radiation risk. Therefore, a review of some ways to accomplish this goal is critical.

One described method to reduce exposure is to lower CT beam intensity, known as low dose FBP-CT. Although fairly reliable, low dose FBP-CT protocols have been found to miss up to 30% of small stones [15], are less effective in obese patients [16], and lead to increased image noise when overly decreased [17]. Moreover, multi-institutional data have shown that low dose protocols are rarely utilized for suspected urolithiasis, and, even when implemented, dose varies greatly depending on the center [18]. Newer CT iterative reconstruction techniques, utilized in this study, have been reported to lower patient dose but have not been as widely accepted as low dose FBP-CT, mainly due to concerns over image quality and the cost of equipping a scanner.

In the majority of published kidney stone studies, CT dose is reported in terms of scanner radiation output related to energy imparted to a reference phantom (called CTDI_VOL_, typically a 32-cm diameter phantom in adult body imaging) or as DLP, dose length product (mGy-cm), which is CTDI_VOL_ multiplied by total centimeters covered by the scan [19]. As a result, CT dose to a small patient (i.e., a patient with an average diameter less than 32 cm) may be underestimated while larger patients (diameters > 32 cm) may be overestimated. We were able to provide both patient-size and organ-specific absorbed radiation doses for each individual in our study, rather than just reporting scanner output metric of CTDI_VOL_ or estimated radiation doses based on phantom studies [6, 20, 21]. This type of granular inspection, using formulas that were validated in cadavers in the exact same scanners, is the first of its kind in the stone field and allows us to see which organs benefitted the most from IR-CT. Utilizing protocols that optimized each reconstruction algorithm, we also found that total CT reported CTDI_VOL_ dose was a fair surrogate of absorbed organ dose. For instance, colon dose was estimated to be about 5 mGy (17%) lower using our FBP-CT cadaveric OSD estimates (24.0 ± 5.76) compared to CT CTDI_VOL_ (28.7 ± 10.1, Table 3). Conversely, skin, which one would expect to have higher entrance and absorbed dose, was calculated to be about 15% higher in both FBP-CT and IR-CT groups compared to CTDI_VOL_.

In addition to demonstrating that IR-CT lowers acquisition parameters (and therefore dose), we also assessed the impact of imaging technique on radiologist confidence, image quality, and organ specific dose for all stone formers in a blinded fashion. We found no significant differences in image noise or subjective image quality between IR-CT and FBP-CT techniques using appropriately powered, comparable cohorts of stone formers with a range of BMI and stone sizes. These cohort similarities may have occurred because the technical parameters were previously set to optimize the reconstruction algorithm used. In a clinical setting, however, this is exactly how radiologists and institutions set up CT scanning protocols. With these in place, we found that IR-CT protocols allowed for less noise at the same kVp (120), tube-current modulation, and slice thickness. With all these in place, IR-CT exams were comparable to traditional FBP-CT studies in respect to making a stone diagnosis and treatment decisions.

This type of retrospective analysis has some limitations. First, each patient did not undergo both FBP- and IR-CT exams for direct comparison. Approval for this type of study design would be difficult, as the ethics of such a study are disputable. Second, we only included patients with positive findings, limiting our ability to compare diagnostic abilities between the two reconstructive technique types. We do attempt to control for this possibility by our method of randomly assigning patients into CT scanners. Third, we do not evaluate the ability of IR-CT to make other types of abdominal or pelvic diagnoses, a dilemma that occurs often in urgent care or emergency room settings where the differential diagnosis for pain may be wide. Finally, we cannot comment on the ability of either modality to prevent missing a diagnosis, including incidental findings.

## 5. Conclusion

In our cohort of 340 confirmed stone formers, patients who underwent IR-CT exams were exposed to 50-60% less radiation (skin and internal organs) compared to patients in the FBP-CT cohort. With similar image quality and noise, low dose CT, utilizing reconstructive algorithms such as IR, should replace FBP-CT as the standard of care in CT imaging of urinary stones. Urologists and radiologists must be familiar with the CT technology and reconstruction techniques being used on their patients at their center and advocate for radiation reducing protocols.

## Figures and Tables

**Table 1:** Patient demographics and imaging history between modalities.

	FBP-CT (n=250)		IR-CT		p value
			(n=90)		
Male gender (%)	133	(53)	45	(50)	p = 0.61

Mean age (range)	53.4 (18-92)		50.2 (19-92)		p = 0.31

BMI (SD)	30.1	(3.2)	30.5 (2.9)		p = 0.73

Race (%)					
Caucasian	198	(79)	71	(79)	p = 0.95
African American	37	(15)	10	(11)	p = 0.28
Hispanic	13	(5)	5	(6)	p = 0.89
Asian	1	(1)	1	(1)	p = 0.45
Unknown	1	(1)	3	(3)	p = 0.18

Recent*∗* CT (SD)	2.5	(0.5)	1.9	(0.4)	p = 0.15

Key: CT, computed tomography; FBP, filtered back projection; IR, iterative reconstruction; SD, standard deviation.

*∗*: “Recent” CT refers to the mean number of abdominal CT scans performed at our institution over the previous five years, excluding the present imaging.

**Table 2:** CT stone, noise, and quality characteristics between modalities.

	FBP-CT		IR-CT		p value
	(n=250)		(n=90)		
+ Kidney Stone*∗* (%)	221	(88)	80	(89)	p = 0.9
Mean stone diameter, mm (SD)	5.8	(1.3)	5.1	(1.1)	p = 0.3

Kidney Stone Morphology (%)					p = 0.75
Single	192	(87)	73	(91)	
Confluence	16	(7)	4	(5)	
Partial staghorn	3	(1)	1	(1)	
Full staghorn	10	(5)	2	(3)	

+ Ureteral Stone (%)	53	(22)	28	(31)	p = 0.07
Mean stone diameter, mm (SD)	4.7	(1.0)	4.3	(0.9)	p = 0.4

+ Hydronephrosis (%)	48	(22)	24	(30)	p = 0.14

+ Bladder Stone (%)	13	(5)	7	(8)	p = 0.37
Mean stone diameter, mm (SD)	8.5 (2)		3.5	(0.8)	p = 0.09

Noise† (%)					p = 0.23
Minimal	188	(75)	61	(68)	
Diagnostic	60	(24)	29	(32)	
Excessive	2	(1)	0	(0)	

Quality† (%)					p = 0.39
Excellent	141	(56)	42	(47)	
Good	78	(31)	34	(38)	
Acceptable	29	(12)	13	(14)	
Suboptimal	1	(1)	1	(1)	
Poor/Non-diagnostic	1	(1)	0	(0)	

Key: CT, computed tomography; FBP, filtered back projection; IR, iterative reconstruction.

SD, standard deviation; *∗*: 16 patients had kidney and one other stone location; † = interrater reliability (AC2) was high: AC2 for noise was 0.79, and AC2 for quality was 0.87.

**Table 3:** CT radiation dose estimates and differences between modalities.

	FBP-CT	IR-CT	(%)	p value
	(n=250)	(n=90)		
OSD Stomach (mGy)	24.2 ± 6.14	9.33 ± 4.08	18.1 (62)	p < .001
OSD Small bowel (mGy)	22.9 ± 5.28	9.07 ± 4.01	13.8 (60)	p < .001
OSD Colon (mGy)	24.0 ± 5.76	9.3 ± 4.16	14.7 (61)	p < .001
OSD Skin (mGy)	34.4 ± 8.83	15.7 ± 11.2	18.7 (54)	p < .001
CTDI_VOL_ dose (mGy)	28.7 ± 10.1	13.7 ± 12.1	15.0 (52)	p < .001

Key: CT, computed tomography; FBP, filtered back projection; IR, iterative reconstruction; SD, standard deviation; OSD: organ specific dose; CTDI_VOL_: measured volumetric CT dose index.

## Data Availability

All data from this study is secured within an institutional database repository (REDCaP). As required by our Institutional Review Board, all data will be purged from the system and destroyed after five years. Thus, readers will not be given access to this particular study data.

## Supplementary Materials

Supplementary Materials Supplemental Figure 1: effective body diameter (EBD) calculation: the image depicts how a lateral and anterior-posterior diameter of a patient's CT can be taken to determine the elliptical area of a patient's cross section. This allows for a calculation of the patient's EBD, which is the effective diameter of a circle with equal area to that of the elliptical cross section on the CT.
