# Supplementary figures and images for: Image Quality and Patient-Specific Organ Doses in Stone Protocol CT: A Comparison of Traditional CT to Low Dose CT with Iterative Reconstruction
Source: Biomed Res Int. 2018 Sep 27;2018:5120974. doi: 10.1155/2018/5120974 (PMC6181004; doi:10.1155/2018/5120974)

Supplementary Figure 1

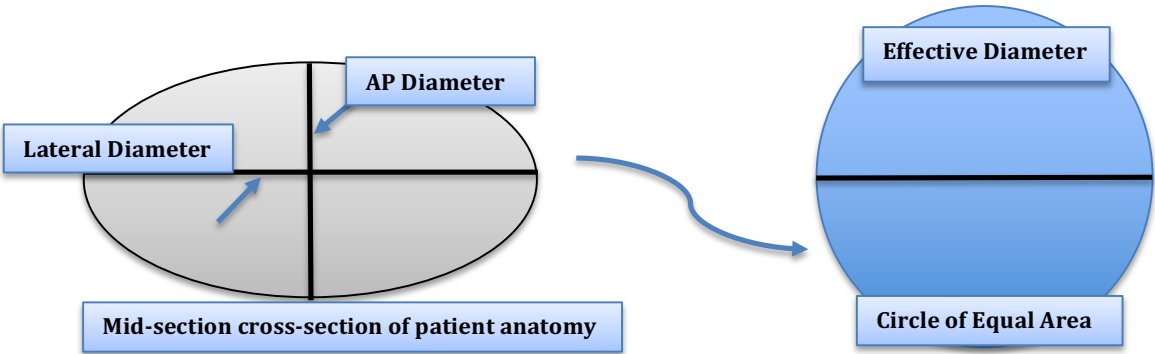

Supplement: Supplementary Materials — Supplemental Figure 1: effective body diameter (EBD) calculation: the image depicts how a lateral and anterior-posterior diameter of a patient's CT can be taken to determine the elliptical area of a patient's cross section. This allows for a calculation of the patient's EBD, which is the effective diameter of a circle with equal area to that of the elliptical cross section on the CT. [file 5120974.f1.pdf]
